# A missense mutant of *ocrl1* promotes apoptosis of tubular epithelial cells and disrupts endocytosis and the cell cycle of podocytes in Dent-2 Disease

**DOI:** 10.1186/s12964-023-01272-4

**Published:** 2023-12-05

**Authors:** Limin Huang, Yingying Zhang, Haidong Fu, Weizhong Gu, Jianhua Mao

**Affiliations:** 1 Department of Nephrology, Children’s Hospital, Zhejiang University School of Medicine. National Clinical Research Center for Child Health, Hangzhou, China; 2 Department of Pediatrics, Clinical Center of Pediatric Nephrology of Henan Province, The First Affiliated Hospital of Zhengzhou University, Zhengzhou, China; 3 Department of Pathologyology, Children’s Hospital, Zhejiang University School of Medicine. National Clinical Research Center for Child Health, Hangzhou, China

**Keywords:** Lowe syndrome, Dent disease, Whole exome sequencing, Podocytes, Cell cycle, Renal tubular, Reactive oxidative species

## Abstract

**Background:**

This study aimed to identify an *orcl1* mutation in a patient with Dent-2 Disease and investigate the underlying mechanisms.

**Methods:**

The *ocrl1* mutation was identified through exome sequencing. Knockdown of *orcl1* and overexpression of the *orcl1* mutant were performed in HK-2 and MPC5 cells to study its function, while flow cytometry measured reactive oxygen species (ROS), phosphatidylserine levels, and cell apoptosis. Scanning electron microscopy observed crystal adhesion, while transmission electron microscopy examined kidney tissue pathology. Laser scanning confocal microscopy was used to examine endocytosis, and immunohistochemical and immunofluorescence assays detected protein expression. Additionally, podocyte-specific *orcl1* knockout mice were generated to investigate the role of *orcl1* in vivo.

**Results:**

We identified a mutation resulting in the replacement of Histidine with Arginine at position 318 (R318H) in *ocrl1* in the proband. *orcl1* was widely expressed in the kidney. In vitro experiments showed that knockdown of *orcl1* and overexpression of *ocrl1* mutant increased ROS, phosphatidylserine exocytosis, crystal adhesion, and cell apoptosis in HK-2 cells. Knockdown of *orcl1* in podocytes reduced endocytosis and disrupted the cell cycle while increasing cell migration. In vivo studies in mice showed that conditional deletion of *orcl1* in podocytes caused glomerular dysfunction, including proteinuria and fibrosis.

**Conclusion:**

This study identified an R318H mutation in *orcl1* in a patient with Dent-2 Disease. This mutation may contribute to renal injury by promoting ROS production and inducing cell apoptosis in tubular cells, while disrupting endocytosis and the cell cycle, and promoting cell migration of podocytes.

Video Abstract

**Supplementary Information:**

The online version contains supplementary material available at 10.1186/s12964-023-01272-4.

## Background

Dent Disease is a chronic nephrosis characterized by low molecular weight (LMW) proteinuria, hypercalciuria, kidney stones, and proximal tubular dysfunction leading to adult renal failure [1]. It predominantly affects males in early childhood and may progress to end-stage renal failure. Approximately 60% of cases (Dent-1 Disease) are due to *clcn5* gene mutations encoding CLC-5, an electrogenic Cl^−^/H^+^ exchanger in proximal tubule cells involved in receptor-mediated endocytosis of albumin and LMW proteins. Dent-2 Disease is caused by mutations in *orcl1*, the gene encoding the type II phosphatidylinositol bisphosphate 5-phosphatase [2]. *orcl1* controls multiple biological processes including the endocytic cycle, endosome to Golgi transfer, early endocytosis, phagocytic steps, cytokinesis, and cilia formation [3].

Dent-2 patients with *orcl1* mutations share similar symptoms with Dent-1 patients [4]. Mutations in *orcl1* disturb phosphatidylinositol metabolism, affecting cell signaling and membrane dynamics, leading to dysfunction of kidney proximal tubule cells. This can cause Dent-2 disease or the more severe Lowe syndrome [5]. The Human Gene Mutation Database records around 250 *orcl1* disease-causing mutations, including various types like missense and nonsense mutations, splicing mutations, small deletions, small insertions, and gross deletions and insertions [6]. However, it remains unknown why some *ocrl* mutations lead to Lowe syndrome and others to the milder Dent-2 disease. Dent's disease is associated with the dysfunction of podocytes [7]. Additionally, the malfunction of tubular epithelial cells can trigger symptoms such as hypercalciuria and kidney stones. The progression of Dent's disease is marked by apoptosis in these cells, resulting in tubular atrophy and loss of kidney function [8]. Overproduction of reactive oxygen species (ROS) can induce apoptosis in renal tubular epithelial cells [9]. Moreover, ROS and related inflammation are highly involved in the pathogenesis of kidney stones [10]. The link between persistent asymptomatic isolated haematuria in children and prognosis, as well as the role of Tempol in protecting against acute kidney injury, has been demonstrated in previous studies by the subject group [11, 12]. However, it is still unclear how the loss of function of *ocrl1* leads to the symptoms associated with proximal renal tubular and glomerular disorders. Understanding these mechanisms is vital for developing more effective diagnostic and therapeutic strategies for Dent's disease.

In this study, we aimed to investigate the underlying mechanisms by which *ocrl1* mutation leads to Dent-2 Disease through in vitro and in vivo experiments. We conducted whole exon sequencing (WES) in a Dent-2 Disease patient and identified a missense mutation in *ocrl1*. Our findings revealed that the missense mutation (p.Arg318His) induced apoptosis in tubular epithelial cells and disrupted the cell cycle of podocytes. These results could lead to more precise diagnoses and better therapeutic options for Dent-2 Disease patients in clinical practice.

## Materials and methods

### Clinical samples

This study was approved by the Ethics Committee of The First Affiliated Hospital of Zhejiang University School of Medicine (No2020057), Hangzhou, China. Written informed consent was obtained from the participant and their guardians prior to enrollment. Blood samples were collected on August 27^th^, 2015 at the Department of Nephrology, Zhejiang University Paediatric Hospital. Renal biopsies were performed as part of routine clinical diagnostic investigations to determine the expression of *ocrl1* in the kidneys of both proband and control patients. Renal biopsy samples were obtained from the Department of Pathology, The Children’s Hospital of Zhejiang University School of Medicine. Control samples were collected from the healthy kidney poles of individuals who underwent tumor nephrectomies without renal disease.

### Generation of podocyte-specific OCRL1 knockout mice

The mice study was approved by The Ethics Committee of the Laboratory Animal Center of Zhejiang University (No. 21377). With recent studies on Dent disease, attention has been focused on whether podocyte damage is the result of renal tubular disease or whether defects such as *ocrl1* directly cause glomerulopathy. The purpose of generating mice with a specific knockout of *ocrl1* in podocytes was to investigate whether a single factor of renal tubular disease could also cause primary glomerular damage. *ocrl1*^flox/flox^ mice with a C57BL/6 background were crossed with Podocin-Cre mice to generate podocyte-specific *ocrl1* knockout (Pod-*ocrl1*-KO) mice. *ocrl1*^flox/flox^/Cre(-) and *ocrl1*^+/+^/Cre( +) mice served as controls, while *ocrl1*^flox/flox^ Cre( +) mice were used as conditional knockout (cKO) mice (*n* = 6). The sample size was calculated using the resource equation method [13, 14]. An "E" value that represents the degree of freedom of analysis of variance was calculated using the equation: E = total number of animals—total number of groups. Any sample size that keeps E between 10 and 20 is considered adequate. The total number of animals in our study was 12, and the number of groups was 2, generating an E value of 12—2 = 10. All mice were housed in the Laboratory Animal Center of Zhejiang University under specific pathogen-free conditions, with free access to food and water, and maintained at a temperature of 25 °C and 50% humidity. Control and Pod-*ocrl1*-KO mice were subjected to tail genotyping. Mice were euthanized with 4% chloral hydrate at 8, 12, 16, and 20 weeks. The kidneys of control (*n* = 6) and Pod-*ocrl1*-KO mice (*n* = 6) were harvested.

### Whole exome and RNA sequencing

DNA was extracted from the blood of the proband and his parents using standard protocols. The Roche Nimble Gen Seq EZ exome enrichment kit with capturing probes V2.0 (Roche, Indianapolis, IN, USA) was used to construct the whole-exome library and enriched the DNA of total exons and their flanking introns. High-throughput sequencing was performed on an Illumina NovaSeq 6000 series sequencer (Illumina, San Diego, CA, USA), with a minimum of 99% of target sequences sequenced at a 150 × reading depth. To identify pathways involved in the cell cycle, total RNA from scramble controls and the OCRL knockdown group of MCP5 cells was extracted using TRIzol. Enriched signaling pathways were selected through RNA sequencing, and the relevant genes were validated using RT-PCR. Data were analyzed using GraphPad software.

### Cell culture

HK-2 cells were purchased from the Shanghai Cell Bank, Chinese Academy of Sciences and cultured in RPMI culture medium supplemented with 10% fetal bovine serum (FBS), 100 U/mL penicillin, and 100 μg/mL streptomycin at pH 7.4. The cells were maintained at 37 °C in a humidified atmosphere of 5% CO_2_ and passaged upon reaching an 80–90% confluent monolayer following trypsin digestion. The immortalized mouse podocyte cell line (MPC5) was generously donated by Professor Peter Mundel (Goldfinch Bio.Inc., Cambridge, MA, USA). The MPC5 cell line was originally derived from the podocytes of a male mouse. Although it is not a human cell line, it was used in our research since it is an established and validated model. MPC5 cells were cultured in RPMI medium supplemented with 10% FBS and 10U/mL mouse recombinant interferon-γ (R&D Systems) at 33 ℃ for proliferation, followed by transfer to RPMI medium supplemented with 10% FBS without recombinant interferon-γ for differentiate at 37 ℃ for 10–14 days.

### Knockdown of OCRL1 and overexpression of OCRL1 mutant

Small interfering RNA targeting *ocrl1* (si-*ocrl1*) or *ocrl1* R318H (si-*ocrl1* H318) mutant, as well as lentiviral vectors carrying shRNA against *ocrl1* and adenoviral vectors containing *ocrl1* (R318H) were purchased from Shanghai Ruijing Biological Technology (Shanghai, China), which were used to knockdown *ocrl1* or overexpress the *ocrl1* mutant in HK-2 cells. The targeted sequence of si/shRNA was 5’-ACCGGCAAGCCAAAGTTACCATATTTCTCGAGAAATATGGTAACTTTGGCTTGTTTTTGAATTC-3’. Lentiviral vectors carrying shRNAs 848, 1816, and 2208 targeting *ocrl1* (TRCnumber:0000012848, TRCnumber00000121816, TRCnumber 00000122208; JIMA) were obtained from GenePharma (Shanghai China) to knockdown *ocrl1* in MCP5 cell line. Cells were seeded in 6-well plates at a density of 5 × 10^5^ cells/well, and lentiviral/adenoviral vectors were added to HK-2 cells at MOI of 10 and to MCP5 cells at MOI of 20. To perform cotransfection, shRNA against *ocrl1* was transfected into HK-2 cells. Once the cells reached 60%–70% confluence, transfection with the *ocrl1* mutant was carried out. Puromycin (2 μg/mL) was added 72 h after transfection and maintained for 5–7 days to achieve > 95% transfection efficiency. Scrambled-shRNA was used as a control. MPC5 cells were transfected using the same protocol as for HK-2 cells, except the cells were incubated at 33 °C until transfection efficiency reached 95%. The cells were then transferred to 37 °C for differentiation.

### Western blot analysis

After cell lysis and centrifugation, the supernatant was collected to determine protein concentration using a Pierce BCA Protein Assay kit (#23,225; Thermo Fisher). Equal amounts of protein were then separated by 10% SDS-PAGE and transferred to polyvinylidene difluoride membranes. The membranes were incubated with anti-OCRL1 (#8797; CST, dilution 1:1000), anti-CCND1 (#2922; CST, dilution 1:1000), anti-E2F1 (#3742; CST, dilution 1:1000), anti-CDKN2D (#77,184; CST, dilution 1:1000), anti-GAPDH (#60,004–1-Ig; Proteintech, dilution 1:1000), or anti-β-actin (#3700 l; CST, dilution 1:10,000) antibody overnight at 4 °C. The membranes were then incubated with HRP-conjugated secondary antibodies (#SA00001-1; #SA00001-2; Proteintech, dilution 1:10,000) at 37 °C for 1 h. Signals were detected with ECL solution (#32,209; Thermo Fisher) and visualized with an Odyssey infrared imaging system.

### Immunoprecipitation

Protein-G Dynabeads (Life Technologies) were mixed with anti-OCRL1 (1:200 dilution, CST, #8797), anti-CCND1 (#2922; CST, dilution 1:1000), anti-E2F1 (#3742; CST, dilution 1:1000), or anti-CDKN2D (#77,184; CST, dilution 1:1000) antibodies diluted in phosphate-buffered saline (PBS) with 0.02% Tween. After rotating at 4 °C for 1 h, total cell lysate was added, and the mixture was rotated for 24 h. The proteins were eluted with sample buffer (5 ×) at 96 °C for 5 min, separated by electrophoresis, and detected by immunoblotting with anti-OCRL1, anti-E2F1, anti-CCND1, or anti-CDKN2D antibodies, followed by Alexa Fluor-conjugated secondary antibody (1:10,000 dilution, ProteinTech).

### Flow cytometry

Reactive oxygen species (ROS) and phosphatidylserine levels as well as cell apoptosis were determined by flow cytometry. HK-2 cells were seeded at a density of 1 × 10^5^ cells/mL in 6-well plates. After incubation, the cells were harvested by digestion and centrifugation at 1200 rpm for 5 min. The cells were then resuspended in 500 µL of PBS and stained with 2ʹ,7ʹ-dichlorofluorescein diacetate for ROS measurement. To detect phosphatidylserine eversion, cells were resuspended in 100 μL of binding buffer and incubated with 10 μL of FITC-labeled Annexin-V in the dark for 30 min at room temperature. For cell apoptosis assay, HK-2 cells were digested with 0.25% trypsin (EDTA free), suspended in binding buffer, and incubated with 5 μL Annexin V and 5μL propidium iodide antibodies (#556,547, BD Biosciences) in the dark for 15 min. Cells were analyzed using a Cyto FLEX S flow cytometer (Beckman, USA) and FlowJo software.

### Scanning electron microscopy (SEM)

To observe crystal adhesion on the cell surface, cells were seeded on coverslips in 12-well plates at a density of 1 × 10^5^ cells/mL. After incubation, the cells were washed twice with D-Hanks solution and exposed to a serum-free medium containing 200 μg/mL calcium oxalate monohydrate (COM) for 1 h. The supernatant was removed, and the cells were washed twice with PBS. Next, the cells were fixed with 2.5% glutaraldehyde for 24 h, washed thrice with PBS, and dehydrated with graded ethanol (50%, 70%, 90%, and 100%). The cells were then dried with CO_2_, sprayed with gold, and analyzed by SEM to visualize crystal adhesion.

### Transmission electron microscopy (TEM)

To observe kidney tissue pathology, samples were fixed in 10% formalin for 72 h, embedded in paraffin, and cut into 4-µm sections. For TEM, 1 × 1 × 1 mm sections of the kidney were fixed in 2.5% glutaraldehyde overnight at 4 ℃. TEM images were obtained by the Center of Electron Microscopy, Zhejiang University School of Medicine, while sample handling and detection were performed by the Analysis Center of Agrobiology and Environmental Sciences & Institute of Agrobiology and Environmental Sciences. Results were analyzed using Image J software (NIH, Bethesda, MD, USA) by two investigators blinded to the experimental details.

### Laser scanning confocal microscope

To examine endocytosis, an internalization assay using human serum transferrin Alexa Fluor conjugate (#148,026; Jackson Immune Research) was conducted with some modifications as described [15]. Surface-bound transferrin was removed using citrate buffer (pH 2.5). Images were collected at 4 h post-incubation using a Nikon A1 Ti laser scanning confocal microscope. Cells with punctate transferrin labeling were considered positive, while cells without distinct transferrin puncta were considered negative.

### Immunohistochemistry

Paraffin sections were subjected to a deparaffinization process in a 60 °C oven for 30–60 min, followed by sequential immersion in three changes of xylene for 10 min each and ethanol gradients (100%, 95%, 80%, and 70%) for 2 min each. The sections were then washed with water for 5 min. To reduce endogenous peroxidase activity, the sections were treated with pre-warmed permeation solution for 30 min (protected from light), followed by incubation at 37 °C for 30 min with anti-OCRL1 antibody (#8797; CST, dilution 1:1000) or IgG (1:10000 dilution, ProteinTech). The sections were then stained with DAB-H_2_O_2_ for 10 min and observed under microscopy.

### Immunofluorescence staining

MCP5, HK-2 cells, and frozen kidney sections were fixed with 4% paraformaldehyde and blocked with 5% bovine serum albumin for 1 h at room temperature. After blocking, cells and tissues were incubated overnight at 4 °C with anti-OCRL1 (#8797; CST, dilution 1:1000), anti-podocin (#ab50339; Abcam, dilution 1:1000), or anti-nephrin (#AF3159; R&D Systems, dilution 1:250) antibodies. The following day, the samples were stained with a mixture of Alexa Fluor 488 donkey anti-goat IgG (#A11055; Life Technologies, dilution 1:500) and Alexa Fluor 594 donkey anti-rabbit IgG (#A21207; Life Technologies, dilution 1:500) secondary antibodies at 37 °C for 1 h in the dark. Images were captured using a Nikon A1 Ti laser scanning confocal microscope.

### Transwell assay

MPC5 cells were suspended in serum-free medium and adjusted to a cell density of 1–10 × 10^5^/mL. A 100–200 μL of cell suspension was added to the upper chamber containing serum-free medium, and the medium containing 10% FBS was added to the lower chamber. The cells were then incubated for 12–48 h. After removing the chambers, the medium was aspirated, and the cells in the Matrigel and upper chamber were gently wiped with a cotton swab. The chambers were then fixed with 4% paraformaldehyde (600 μL) in a new 24-well plate for 20–30 min. After discarding the fixative, the cells were stained with 0.1–0.2% crystalline violet for 5–10 min and washed three times with PBS. The samples were then examined under a microscope.

### Statistical analysis

Data were presented as the mean ± standard deviation. Statistical analysis was performed using GraphPad Prism version 8.0. Nonparametric tests were used for comparing two groups. Statistical significance was considered at *P* < 0.05.

## Results

### Clinical presentation of the proband

The proband, a 3-year-old Chinese boy, presented with severe illness and was evaluated for urination at the Children's Hospital of Zhejiang University. In 2015, the patient was diagnosed with proteinuria and developed nephrotic-range proteinuria (65.8 mg/kg) with a urine protein/creatinine of 5.06, α1 microglobulin 276.9 mg/L, and β2 microglobulin 15.4 mg/L. Subsequently, the patient underwent a kidney biopsy which revealed extensive fusion of the foot processes and a basement membrane thickness of approximately 350 nm. Notably, the patient’s parents had no symptoms and normal urine tests. Post-discharge, the patient continued to take fosinopril sodium and hydrochlorothiazide tablets, with urine protein maintained between + and +  + . In 2019, the patient was hospitalized for acute renal failure and improved after dialysis treatment.

### Identification of *ocrl1* variant in proband

**Fig. 1:**
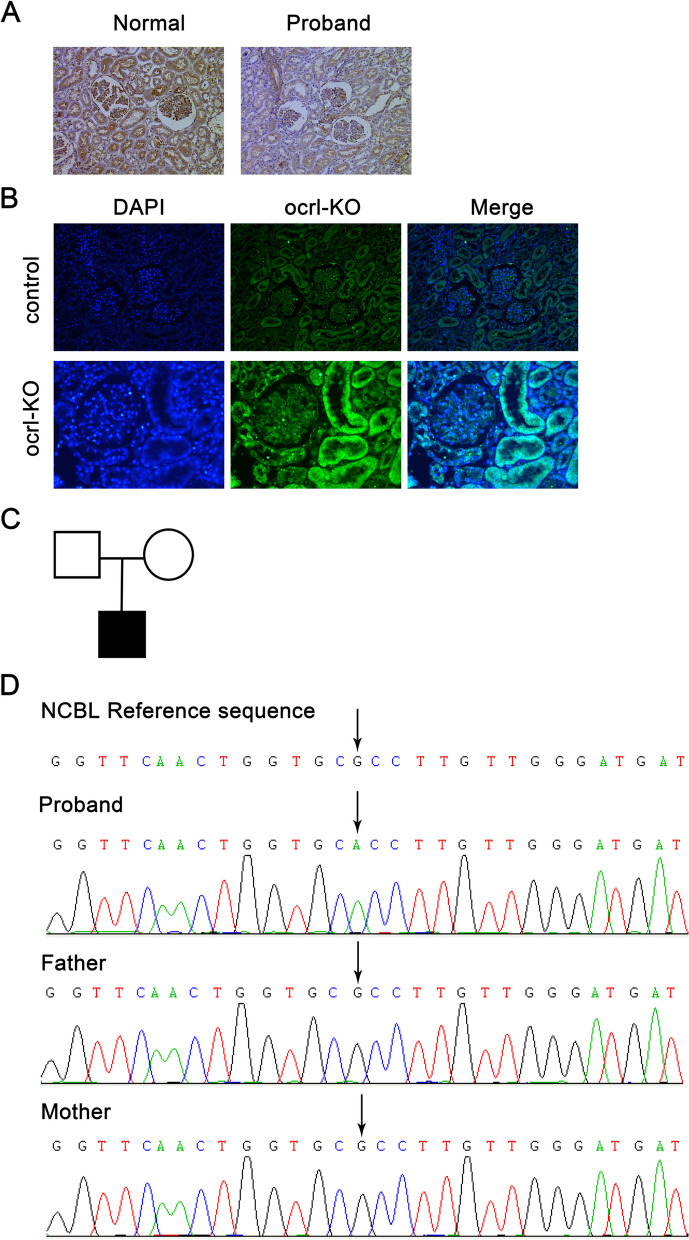
Identification of an OCRL inositol polyphosphate-5-phosphatase (*ocrl1*) mutation in a patient with Dent-2 Disease. **A**, Immunohistochemical staining shows minimal changes in nephropathy, and *ocrl1* expression was observed in the tubules and glomeruli of the proband’s kidney. **B**, *ocrl1* expression in the mouse kidney was assessed using immunofluorescence. **C**, Pedigree of the proband's family. Empty symbols represent normal individuals. Filled square indicates the proband. **D**, Whole exome sequencing identified a mutation resulting in the replacement of Histidine with Arginine at position 318 in *ocrl1* in the proband. KO, knockout

WES was performed on the proband and his parents, which identified a variant on the X chromosome of the proband: *ocrl1* (NM_128696374: c.953G > A, E11). The patient’s parents did not have this variant (Fig. 1C and D). The mutation resulted in the replacement of Histidine with Arginine at position 318 (R318H, *ocrl1* variant 1 NM_128696374) in the shorter variant. Additionally, another variant was identified in the proband: CFB (NM_001710.6: c.1343A > G (E10)) and will be investigated in the future.

### In situ expression of OCRL1 in normal and diseased kidneys

Immunohistochemical analysis was performed on histological sections of the proband’s renal tissue to compare in situ expression of *ocrl1* with a normal kidney biopsy sample from a patient who underwent tumor nephrectomies without renal disease (Fig. 1A). Immunofluorescence of OCRL1 protein in mouse kidneys was also detected (Fig. 1B). The results showed that OCRL1 protein was present in all cell types within the glomerulus (mesangial cells, endothelial cells, and podocytes) and renal tubular cells in both the normal kidney and the proband's kidney, but barely detectable in the kidney of Pod-*ocrl1*-KO mice. No difference in spatial divisions was observed.

### *ocrl1* regulates ROS production, apoptosis and crystal-cell adhesion in renal tubular cells

**Fig. 2:**
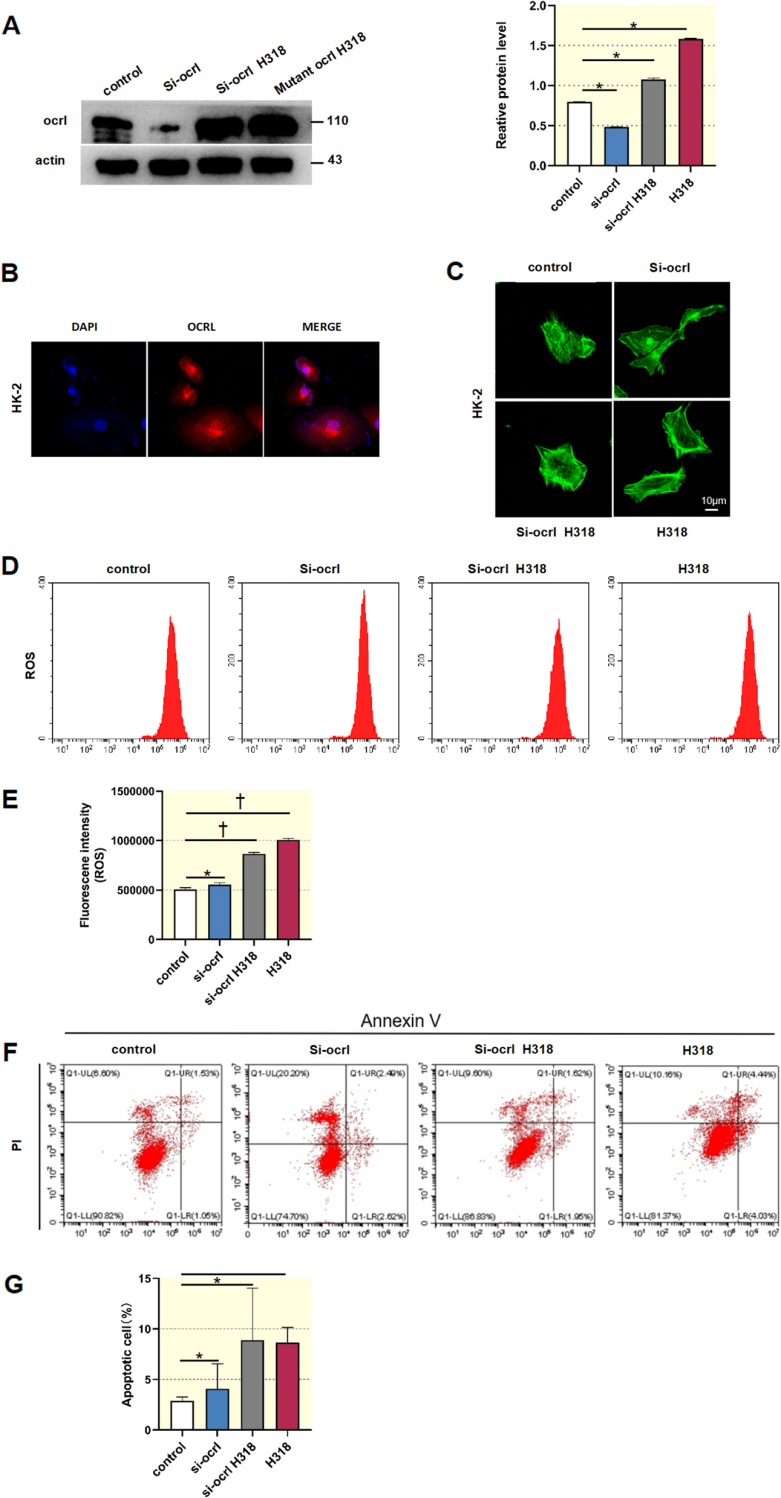
Knockdown of *ocrl1* and overexpression of the *ocrl1* mutant induced reactive oxygen species (ROS) production and apoptosis in HK-2 cells. **A**, HK-2 cells were transfected as indicated. The protein level of *ocrl1* was determined by Western blot analysis (*n* = 3). **B**, Confocal microscopy demonstrated that the *ocrl1* protein is primarily localized in the cytoplasm of HK-2 cells. **C**, Confocal microscopy showed that knockdown of *ocrl1* and overexpression of *ocrl1* mutant did not affect the cytoskeleton. **D–G**, Flow cytometry was conducted to assess ROS levels (D, E) and cell apoptosis (F, G) in HK-2 cells. Data were expressed as the mean ± standard error of the mean (SEM). *P < 0.05, †*P* < 0.01 vs. control; *n* = 3. si-*ocrl1*, siRNA targeting *ocrl1*; H318, adenoviral vectors overexpressing *ocrl1* mutant

To investigate the role of *ocrl1* and its mutant in renal tubular cells, we transfected HK-2 cells with si-*ocrl1*, si-*ocrl1* H318, or adenoviral vectors for overexpressing *ocrl1* H318. Subsequently, we assessed ROS production, cell apoptosis, and phosphatidylserine eversion. As shown in Fig. 2A, transfection with si-*ocrl1* resulted in a significant reduction of over 50% in OCRL1 protein levels. Conversely, transfection with si-*ocrl1* H318 significantly elevated OCRL1 protein expression compared to the control condition. Furthermore, overexpression of *ocrl1* H318 led to a further augmentation in OCRL1 protein levels. The OCRL1 protein was primarily localized in the cytoplasm (Fig. 2B). Confocal images demonstrated that knocking down *ocrl1* or overexpressing *ocrl1* mutant did not impact the arrangement of the cytoskeleton (Fig. 2C). Flow cytometry analysis showed that knocking down *ocrl1* significantly increased intracellular ROS levels in HK-2 cells, which were further elevated by overexpression of the *ocrl1* mutant (Fig. 2D and E). Similarly, this trend was observed in HK-2 cell apoptosis (Fig. 2F and G).

**Fig. 3:**
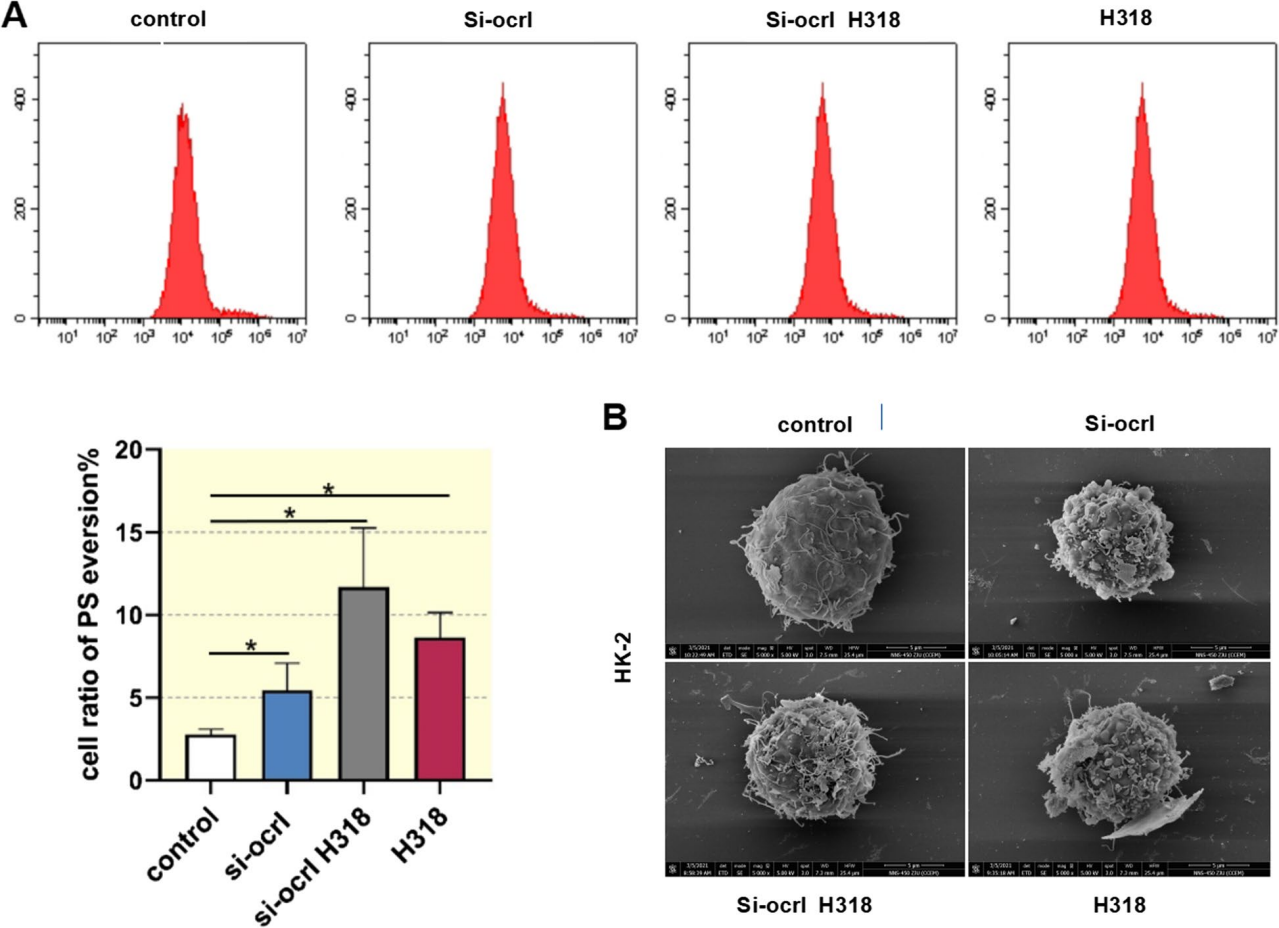
Knockdown of *ocrl1* and overexpression of the *ocrl1* mutant induced phosphatidylserine eversion and crystal adhesion in HK-2 cells. **A**, HK-2 cells were transfected as indicated. Flow cytometry analysis was performed to measure phosphatidylserine eversion. Data were expressed as the mean ± SEM. *P < 0.05 vs. control; *n* = 3. **B**, Scanning electron microscopy was used to examine the adhesion of HK-2 cells to calcium oxalate monohydrate. si-*ocrl1*, siRNA targeting *ocrl1*; H318, adenoviral vectors overexpressing *ocrl1* mutant

To understand the role of *ocrl1* in crystal adhesion, which is critical for kidney stone formation in Dent's disease [16], we evaluated phosphatidylserine eversion in HK-2 cells following *ocrl1* knockdown or overexpression of *ocrl1* mutant. Flow cytometry analysis revealed that in the control group, phosphatidylserine eversion was minimal (2.86%). However, it increased to 4.08% in response to the knockdown of *ocrl1* and further increased in response to si-*ocrl1* H318 and the overexpression of *ocrl1* mutant (8.89% and 8.64%, respectively) (Fig. 3A). SEM images demonstrated the adhesion of HK-2 cells to COM particles (~ 100 nm) following knockdown of *ocrl1* and overexpression of *ocrl1* mutant. The control group had only a few adhered crystals (Fig. 3B). The results suggest that *ocrl1* and its R318H mutation notably influence intracellular ROS levels, cell apoptosis, and phosphatidylserine eversion, with the latter potentially contributing to increased crystal adhesion, a critical aspect of kidney stone formation in Dent-2 disease.

### *ocrl1* deficiency impairs endocytosis and cell cycle transition while promoting cell migration of podocytes

**Fig. 4:**
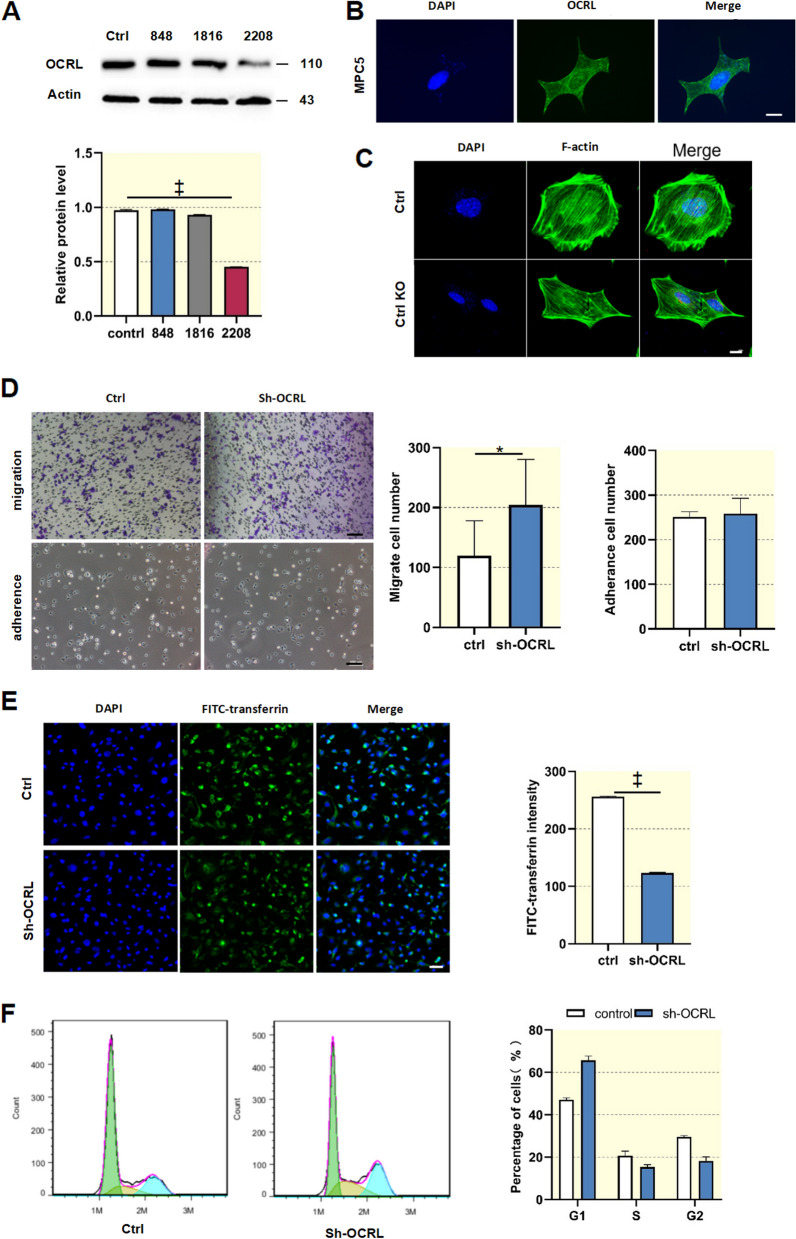
Knockdown of *ocrl1* increased podocyte migration and decreased endocytosis activity. **A**, Mouse MCP5 podocytes were transfected with shRNAs 848, 1816, and 2208 targeting *ocrl1*. Western blot analysis was performed to evaluate knockdown efficiency (*n* = 3). **B**, MCP5 cells transfected with GFP-tagged OCRL1 protein showed a primarily intracellular distribution of green fluorescence. Scale bar: 10 μm. **C**, Confocal microscopy analysis indicated that knockout of *ocrl1* did not impact the structure of microtubules and microfilaments in podocytes, as evidenced by staining for α-tubulin. Scale bar: 10 μm. **D**, Transwell analysis was conducted to assess podocyte migration. Scale bar: 100 μm. **E**, An internalization assay was conducted using Alexa-568-transferrin to evaluate endocytosis in MCP5 cells. The images were captured 4 h after the cells were incubated with transferrin. **F**, Flow cytometry was used to measure the percentage of cells in the S phase, G1 phase, and G2 phase. Data were expressed as the mean ± SEM. *P < 0.05; †*P* < 0.01; ‡*P* < 0 .001 vs. control; *n* = 6

To further investigate whether the loss of *ocrl1* affects podocyte function, an *ocrl1* knockdown podocyte line was generated in vitro through transfecting MPC5 cells with shRNA 2208 against *ocrl1*. As shown in Fig. 4A, transfection with shRNA 2208 resulted in a notable decrease of over 50% in OCRL1 protein levels in MPC5 cells. Given that overexpression of the mutant protein and *ocrl1* silencing in tubular cells produced similar effects with different magnitudes, we opted to perform *ocrl1* silencing exclusively in MPC5 cells. MCP5 cells transfected with GFP-tagged OCRL1 protein showed a primarily cytosolic distribution of green fluorescence, suggesting that OCRL1 protein was predominantly localized in the cytoplasm of podocytes (Fig. 4B). Although *ocrl1* deficiency did not affect the distribution of the actin skeleton in podocytes, it did lead to changes in podocyte morphology (Fig. 4C). Notably, a transwell assay showed that *ocrl1* knockdown cells exhibited an increased cell migration rate compared with control podocytes. This enhanced cell migration rate was deemed abnormal and indicative of podocyte injury. However, the knockdown of *ocrl1* did not affect cell adhesion in podocytes (Fig. 4D).

*ocrl1* is involved in the endocytosis of cells, which plays an important role in maintaining normal cellular function [17]. To investigate the role of *ocrl1* in endocytosis in podocytes, we monitored the uptake of Alexa Fluor-conjugated transferrin. The fluorescence intensity of endocytosed transferrin was significantly lower in *ocrl1* knockdown cells than in control podocytes (Fig. 4E). Furthermore, *ocrl1* deficiency increased podocyte migration and weakened endocytosis without affecting cell adhesion. After *ocrl1* knockdown, the percentage of cells in the G1 phase significantly increased from 46% to 65.94%, while the percentage of cells in the G2 and S phases decreased (Fig. 4F). These findings suggest that *ocrl1* mutation leads to the arrest of podocytes in the G1 phase.

### *ocrl1* deficiency disrupts podocyte function through altered gene expression and protein–protein interactions

**Fig. 5:**
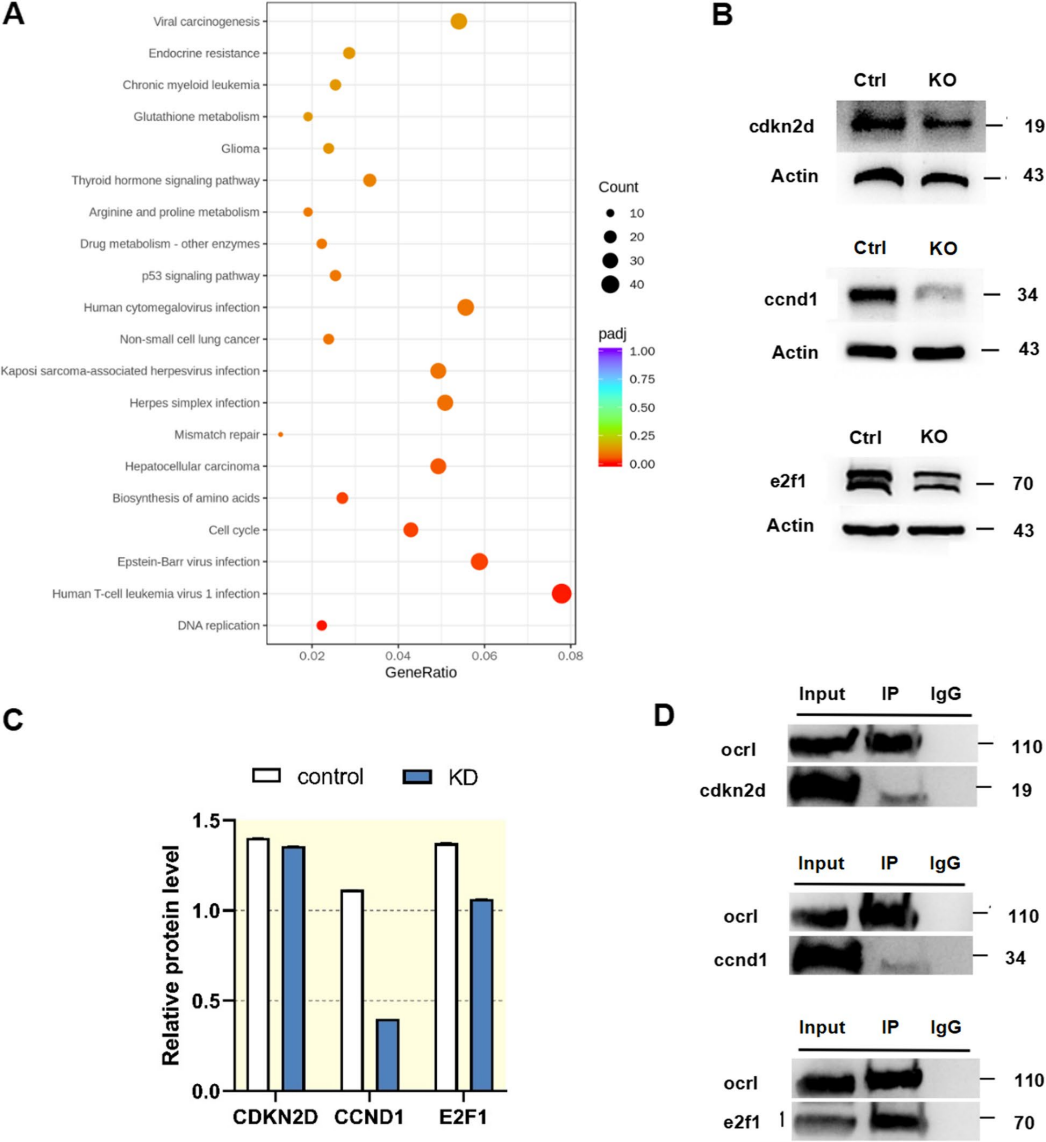
Identification of proteins interacting with OCRL1. **A**, Kyoto Encyclopedia of Genes and Genomes analysis using RNA sequencing data from podocyte cells. **B** and **C**, Western blot analysis demonstrates the effect of *ocrl1* knockdown on the protein levels of the cell cycle pathway in podocytes (*n* = 3). Immunoprecipitation and protein pull-down analysis revealed the interaction between *ocrl1*, CCND1, E2F1, and CDKN2D. **D**, Co-immunoprecipitation experiments were performed using control antibody (IgG) or antibodies to OCRL1, CCND1, E2F1, and CDKN2D to demonstrate the interaction between the endogenous proteins. OCRL1 and E2F1 showed a relatively close interaction

To explore the potential mechanisms underlying the effect of *ocrl1* deficiency on podocyte function, we conducted RNA sequencing and identified differentially expressed genes in *ocrl1* knockdown samples compared to control samples (|fold change|> 1.5), including three downregulated genes (E2F1, CCND1, and CDKN2D). Additionally, the Kyoto Encyclopedia of Genes and Genomes analysis demonstrated that the altered proteins were enriched in the cell cycle pathway (Fig. 5A). Moreover, Western blotting confirmed the reduced expression of E2F1, CCND1, and CDKN2D at the protein level. We performed immunoprecipitation and protein pull-down experiments to investigate the protein–protein interactions of OCRL1 in cultured podocytes. Remarkably, we observed that OCRL1 co-immunoprecipitated with the E2F1 protein in podocytes (Fig. 5B and C). This interaction between OCRL1 and E2F1 was predicted to be critical for maintaining normal podocyte function (Fig. 5D). These findings may help to explain the mechanisms underlying the impaired podocyte function observed in *ocrl1* deficiency.

### Podocyte-specific knockout of *ocrl1* in mice (Pod-OCRL1-KO) results in renal pathological changes

**Fig. 6:**
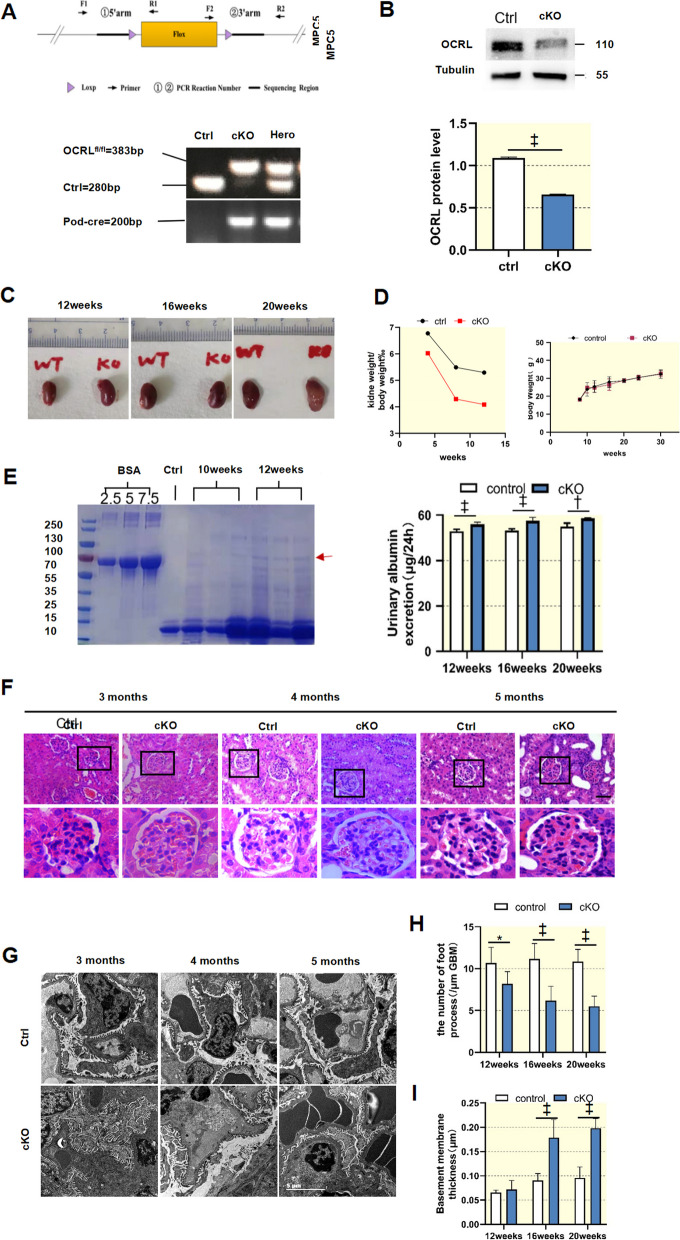
Podocyte-specific knockout of *ocrl1* in mice (Pod-OCRL1-KO) resulted in renal pathological changes. **A**, Podocyte-specific knockout of *ocrl1* was achieved in mice (Pod-*ocrl1*-KO) using the Cre-LoxP recombination system. *ocrl1* and Pod-Cre genotypes were confirmed via tail genotyping, which was performed at 2 weeks of age (*n* = 9). **B**, Western blot analysis was carried out to determine the expression of *ocrl1* in control and conditional knockout (cKO) glomerular podocytes. The results showed decreased expression of *ocrl1* in primary glomerular cell cultures from both control mice and Pod-*ocrl1*-KO mice (*n* = 3). **C**, Representative images of kidneys from control and Pod-*ocrl1*-KO mice at 12, 16, and 20 weeks of age. **D**, No significant differences were observed in body weight between Pod-*ocrl1*-KO mice (*n* = 10) and control mice (*n* = 10). **E**, Coomassie blue-stained SDS-PAGE was performed on urine samples obtained from 10-week-old and 12-week-old mice, followed by densitometry quantification of albumin levels (*n* = 3). Additionally, 2.5, 5, and 7.5 μg of BSA were loaded as the positive control (first three lanes; molecular weight, 66.5 kDa; arrowhead). Urinary albumin excretion was quantified in control and Pod-*ocrl1*-KO mice at different ages (*n* = 10). **F**, Representative images of kidney sections stained with haematoxylin and eosin are shown for control and Pod-*ocrl1*-KO mice. Glomerular fibrosis (indicated by arrowheads) was observed in the kidneys of cKO mice at 5 months of age when stained with haematoxylin and eosin. Scale bar: 50 μm. **G**, Representative images obtained through transmission electron microscopy at different ages in mice. **I**,** J**, Quantification was performed to determine the mean foot process width and the number of foot processes in Pod-*ocrl1*-KO mice at 3, 4, and 5 months of age. *ocrl1*^flox/flox/Cre(−)^ and *ocrl1*.^+/+/Cre(+)^ mice were used as controls. Scale bar: 5 μm. *P < 0.05; †*P* < 0.01; ‡*P* < 0.001

To investigate the role of *ocrl1* in podocytes in vivo, we generated Pod-*ocrl1*-KO mice using the Cre-loxP strategy (Fig. 6A). The absence of *ocrl1* in Pod-*ocrl1*-KO mice was confirmed by Western blotting (Fig. 6B). Control mice lacking the Cre gene (*ocrl1*^flox/flox^ /Cre(-)) or lacking the flox gene (*ocrl1*^+/+^/Cre( +)) were used, and all mice were born at Mendelian ratio and appeared normal (Fig. 6C and D). Pod-*ocrl1*-KO mice exhibited mild proteinuria as detected by Coomassie brilliant blue staining from 12 weeks and an increase in urine albumin compared with control mice (Fig. 6E). Renal pathological characteristics were not significantly different between Pod-*ocrl1*-KO mice and controls at 16 weeks (Fig. 6F), but glomerular fibrosis appeared at 20 weeks (Fig. 6F). Additionally, glomerular ultrastructure changes, such as foot process broadening and effacement, were present from 16 weeks and were not seen in control mice. Pathological histology revealed significant increases in basement membrane thickness in Pod-*ocrl1*-KO mice (Fig. 6I and J). These results indicate that the absence of *ocrl1* in podocytes leads to pathological changes and highlights the importance of this protein in maintaining normal renal function.

## Discussion

In this study, we described the clinical presentation of a 3-year-old boy diagnosed with Dent-2 Disease and the identification of a pathogenic missense mutation in the *ocrl1* gene. This mutation has also been recently reported by Lee et al. [18] We examined the role of *ocrl1* in regulating ROS production, apoptosis, and crystal-cell adhesion in renal tubular cells and its impact on podocyte function through altered gene expression and protein–protein interactions. Additionally, we generated Pod-*ocrl1*-KO mice to demonstrate the importance of *ocrl1* in maintaining normal renal function. Our findings highlight the crucial role of *ocrl1* in renal function and its potential implications for the diagnosis and treatment of renal diseases.

Dent Disease exhibits genetic and phenotypic heterogeneity, leading to several different clinical classifications, but LMW proteinuria, hypercalciuria, and nephrocalcinosis/nephrolithiasis remain hallmark pathologies. In general, total protein excretion associated with tubular proteinuria in Dent Disease is lower than that of glomerular diseases [19–21]. This proteinuria typically includes β2 microglobulin, α1 microglobulin, and retinol-binding protein. However, in this study, the patient diagnosed with Dent-2 Disease and harboring a point mutation in *ocrl1* presented atypical nephrotic-range proteinuria with an LMW protein-albumin, indicating a glomerular disorder. Electron microscopy revealed extensive foot process fusion and a basement membrane thickening in the patient's renal biopsy. The unusual presentation prompts consideration of two potential mechanisms for proteinuria pathogenesis in Dent Disease: either diminished reabsorption of physiologically filtered proteins, including albumin, or the presence of glomerular sclerosis suggesting an additional glomerulopathy [19–21]. The primary or secondary nature of the glomerular damage is a significant area of investigation, as substantial proteinuria in Dent Disease patients may suggest primary podocyte damage.

To observe the expression of OCRL1 protein in the kidney tissue under pathological conditions, we performed immunohistochemistry and immunofluorescence staining. We found that OCRL1 was widely expressed in the kidney. To investigate *ocrl1*-mediated renal tubular cell damage, we generated a human HK-2 cell line with *ocrl1* knockdown or *ocrl1* mutant overexpression. Our study revealed that *ocrl1* knockdown or mutant overexpression in HK-2 cells led to ROS production, cell apoptosis, and phosphatidylserine eversion. Elevated ROS can lead to DNA damage, potentially resulting in cell cycle arrest and altered apoptosis protein expression [22]. These findings emphasize the importance of *ocrl1* in maintaining renal tubular integrity and function.

Phosphatidylserine is a critical adhesion molecule usually present in the tubular epithelial cell membrane [23]. However, cell damage causes phosphatidylserine to flip to the cell surface, increasing the adhesive properties of the cells [24]. Kidney stones are mainly composed of COM [25], and the retention of COM crystals is critical to stone formation [26]. Damaged cells show increased expression of adhesion molecules such as hyaluronic acid, phosphatidylserine, eight transmembrane proteins, and osteopontin, promoting more crystals to attach [27]. This aligns with the SEM observations of COM adhesion on renal tubular cells, suggesting a potential explanation for kidney stone symptoms in Dent Disease patients. Even though apoptotic cells are destined for elimination, during their apoptosis process, the increased exposure to phosphatidylserine can lead to greater crystal adhesion. These crystals can act as a foundation for further crystal aggregation, potentially leading to kidney stone formation. Macrophages phagocytose and digest a small number of crystals. However, many crystals aggregate into a mass containing osteopontin and epithelial cell debris and are then excreted into the renal tubular lumen. There, they become the nuclei of urinary stones [28].

To investigate the role of *ocrl1* in glomerular cells, we generated an *ocrl1* knockdown MCP5 podocyte cell line. Our results indicate that *ocrl1* plays an important role in podocyte endocytosis and migration. The podocyte skeleton is essential in maintaining these functions, and changes in actin filaments have been reported in fibroblasts from patients with Dent Disease [29]. This abnormality is thought to be related to the interaction of *ocrl1* with collagen involved in actin remodeling [30]. However, our in vitro study showed no significant changes in podocyte F-actin cytoskeleton after *ocrl1* knockdown in the absence of external factors. This suggests that cytoskeleton changes in podocytes may require the participation of other molecules or mechanisms. It has been reported that OCRL is involved in regulating actin cytoskeleton dynamics. OCRL mutation could disrupt this process, thereby inducing glomerular damage [31]. The discrepancy could be due to the lack of external factors in the in vitro study. Thus, further research is necessary to fully understand the conditions under which *ocrl1* impacts podocyte function.

One important finding of the present study is the altered cell cycle of podocytes following knockdown of *ocrl1*. Podocyte dysfunction, closely linked to proteinuria and nephrotic syndrome, is significantly impacted by cell cycle dysregulation [32, 33]. However, the role of *ocrl1* in podocyte cell cycle regulation, as well as the specific nature of cell cycle dysregulation in podocytes in the context of Dent-2 disease, has yet to be documented. Our study demonstrated a link between *ocrl1* knockdown and decreased expression of proteins CDKN2d, E2F1, and CCND1, as evidenced by Western blot analysis. The reduced expression of CCND1, a key player in cell cycle regulation, especially during the transition from G1 to S phase [34, 35], might potentially disrupt the normal functioning of podocytes. This connection, underscored by co-immunoprecipitation and protein pull-down analyses, has led us to speculate that OCRL1 and CCND1 may interact indirectly, possibly via other molecules. These findings provide novel insights into the role of *ocrl1* in podocyte cell cycle regulation, specifically in the context of Dent-2 disease.

To explore the role of *ocrl1* in podocytes in vivo, we utilized a Pod-*ocrl1*-KO mouse model. The absence of *ocrl1* in podocytes resulted in podocyte dysfunction and proteinuria. Proteinuria in the Pod-*ocrl1*-KO mice became apparent at 12 weeks. Glomerular fibrosis was observed at 20 weeks. This observation is consistent with previous reports, which indicate that glomerular changes in Lowe syndrome include glomerular fibrosis and basement membrane thickening [36, 37].

This study presents an insightful investigation of *ocrl1* mutation's impact on Dent-2 disease, exploring its effects on renal tubular cells and podocytes. Further elucidation is required to establish a correlation between the cellular effects and the overall clinical manifestations associated with Dent-2 disease. There are several limitations that should be acknowledged, including a lack of full understanding of the underlying molecular and signaling pathways impacted by *ocrl1* mutation and a gap in the translation of the murine model findings to the human disease context. Additionally, *Inpp5b*, the paralog of *OCRL*, was not evaluated in our study. The absence of this information limits a comprehensive understanding of the interplay between *OCRL* and *Inpp5b* and its potential impact on the observed phenotypes. Future directions should aim to elucidate the molecular mechanisms further and validate the findings in the human disease setting for a more comprehensive understanding and novel therapeutic strategies.

In conclusion, this report describes a case of Dent-2 Disease in a child with a rare R318H mutation, leading to nephrotic-range proteinuria. The study emphasizes the crucial role of *ocrl1* in maintaining the functional integrity of the renal tubule and glomeruli. Specifically, it highlights the involvement of *ocrl1*-mediated podocyte injury in the regulation of glomerular dysfunction in Dent-2 Disease, potentially through the activation of cell cycle signaling. The findings suggest a direct contribution of *ocrl1* deficiency to the development of glomerulopathy. Further research is needed to fully understand the underlying mechanisms and explore potential therapeutic interventions for Dent-2 Disease.

## Data Availability

All data generated or analysed during this study are included in this published article.

## Abbreviations

ROS: Reactive oxygen species
LMW: Low molecular weight
OCRL: Ophthalmoencephali-renal syndrome
WES: Whole exon sequencing
cKO: Conditional knockout
FBS: Fetal bovine serum
MPC5: Mouse podocyte cell line
COM: Calcium oxalate monohydrate
